# Visualisation and Quantification of Morphogen Gradient Formation in the Zebrafish

**DOI:** 10.1371/journal.pbio.1000101

**Published:** 2009-05-05

**Authors:** Steven A Harvey, James C Smith

**Affiliations:** 1 Wellcome Trust/CR-UK Gurdon Institute and Department of Zoology, The University of Cambridge, Cambridge, United Kingdom; 2 MRC National Institute for Medical Research, The Ridgeway, Mill Hill, London, United Kingdom; Wellcome Trust Sanger Institute, United Kingdom

## Abstract

During embryonic development, signalling molecules known as morphogens act in a concentration-dependent manner to provide positional information to responding tissues. In the early zebrafish embryo, graded signalling by members of the nodal family induces the formation of mesoderm and endoderm, thereby patterning the embryo into three germ layers. Nodal signalling has also been implicated in the establishment of the dorso-ventral axis of the embryo. Although one can infer the existence of nodal gradients by comparing gene expression patterns in wild-type embryos and embryos in which nodal signalling is diminished or augmented, real understanding can only come from directly observing the gradients. One approach is to determine local ligand concentrations in the embryo, but this is technically challenging, and the presence of inhibitors might cause the effective concentration of a ligand to differ from its actual concentration. We have therefore taken two approaches to visualise a direct response to nodal signalling. In the first, we have used transgenic embryos to study the nuclear accumulation of a Smad2-Venus fusion protein, and in the second we have used bimolecular fluorescence complementation to visualise the formation of a complex between Smad2 and Smad4. This has allowed us to visualise, in living embryos, the formation of a graded distribution of nodal signalling activity. We have quantified the formation of the gradient in time and space, and our results not only confirm that nodal signalling patterns the embryo into three germ layers, but also shed light on its role in patterning the dorso-ventral axis and highlight unexpected complexities of mesodermal patterning.

## Author Summary

One of the earliest events in vertebrate embryonic development is the patterning of the embryo into three germ layers: the ectoderm, mesoderm, and endoderm. Morphogens are signalling molecules that act in a concentration-dependent manner to induce the formation of different cell types. Members of the nodal family are thought to form a morphogen gradient in the developing zebrafish embryo and to be essential for pattern formation. Mesoderm and endoderm are believed to develop due to high levels of nodal signalling, while cells experiencing the lowest concentrations of nodal signalling become ectoderm. Although this idea is widely accepted, the formation of a nodal morphogen gradient has never been observed directly, and we have therefore used two different approaches to visualise the intensity of nodal signalling within individual cells. Our approaches have allowed us to visualise a gradient of nodal signalling activity in the developing zebrafish embryo. Quantification of the levels of nodal signalling experienced by individual cells confirms that nodal signalling patterns the animal-vegetal axis of the zebrafish embryo and, in contrast to previous studies, also suggests that it plays a role in patterning the dorso-ventral axis of the zebrafish embryo.

## Abstract 3

Gradients of nodal signalling in developing zebrafish embryos are visualized using a novel biofluorescence complementation reporter and quantified, demonstrating a role for nodal signalling in dorso-ventral patterning in addition to specifying the animal-vegetal axis.

## Introduction

During embryonic development, secreted molecules known as morphogens generate a concentration gradient of positional information that instructs developing tissues to adopt particular cell fates [1]. One example of this phenomenon is the patterning of the early zebrafish embryo by the nodal/TGF-β signalling pathway [2]. Thus, the zebrafish nodal ligands squint (sqt) and cyclops (cyc) are expressed in the most marginal cells of the developing zebrafish embryo and homozygous mutations in both *sqt* and *cyc* result in embryos that lack all endoderm and mesoderm, apart from a few somites in the tail [3]. Similar phenotypes are achieved through the loss of nodal signalling by mutation of both maternal and zygotic *one-eyed-pinhead* (MZ*oep*) or by misexpression of the nodal antagonist *lefty* [4,5].

Misexpression of *sqt* and *cyc* in the zebrafish animal pole indicate that cyc acts only over short distances, whereas sqt functions as a morphogen and exerts its effects over long distances to induce target gene expression [2]. High levels of nodal signalling activate *goosecoid* (*gsc*) expression, whereas lower levels activate *no-tail* (*ntl*). Thus, *gsc* is expressed in cells near a source of sqt, and *ntl* is expressed in cells further away.

The correct regulation of *ntl* is essential for patterning of the zebrafish embryo, because homozygous mutations in *ntl* disrupt mesoderm and notochord formation [6]. The same tissues are disrupted in *Xenopus* embryos lacking *Brachyury* (*Xbra*) [7], and ectopic expression of *Xbra* in isolated animal regions converts ectodermal cells into a mesodermal fate [8]. Consistent with the requirement of nodal signalling for mesoderm formation, *ntl* expression fails to initiate in embryos with diminished nodal signalling [3–5].

Together, these experiments suggest that nodal family members form a gradient that induces target gene expression and specifies mesoderm and endoderm. Activation of the nodal signalling pathway within a cell results in the phosphorylation of Smad2, which then interacts with Smad4 [9]. The resulting Smad2/4 complex translocates to the nucleus where it activates the transcription of target genes. To visualise the formation of a nodal gradient, we have first made use of transgenic embryos expressing a Smad2-Venus fusion protein under the control of a ubiquitous promoter: nodal signalling causes such constructs to enter the cell nucleus [10]. In addition, however, we have exploited the greater signal-to-noise ratio afforded by the technique of bimolecular fluorescence complementation (BiFC) [11]. In this approach, the N- and C-terminal halves of a fluorescent protein are brought into proximity by interactions between the two unrelated proteins to which they are fused. They can then assemble into a functional fluorescent protein that can be detected by conventional microscopy. This approach has previously been used to visualise, in a quantitative manner, interactions between Smad2 and Smad4 in the *Xenopus* embryo [12]. Yolk autofluorescence in *Xenopus* prevented a proper study of endogenous signalling events [12], but in this Research Article, we show that the technique is effective in the zebrafish embryo.

Our data allow us to follow in space and time the formation of a gradient of nodal signalling activity within the developing zebrafish embryo. The results illustrate the dynamics of gradient formation, and in contrast to previous studies [13], clearly demonstrate a role for nodal signalling in dorso-ventral patterning, explaining why target genes such as *gsc* are only expressed in dorsal marginal cells. Our data also highlight the complexities of *ntl* regulation and of the formation of the border between dorsal mesendoderm and the neural plate.

## Results

### Smad2/4 BiFC and Smad2-Venus Act as Sensors of Nodal Signalling Levels

**Figure 1:**
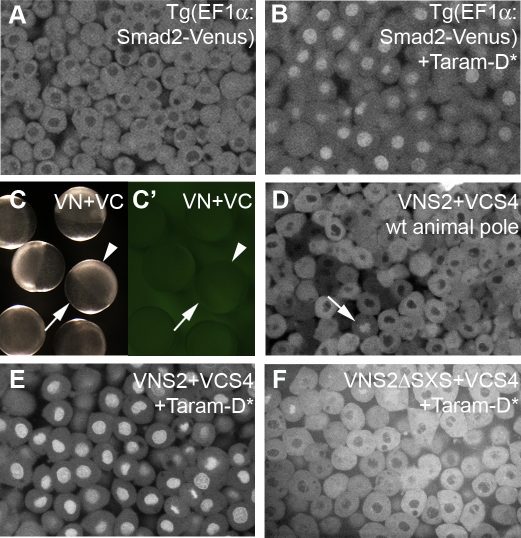
Activation of TGF-β Signalling Results in Nuclear Accumulation of Smad2/4 BiFC and Smad2-Venus (A and B) Animal pole cells of 6-hpf transgenic zebrafish embryos that ubiquitously express a Smad2-Venus fusion protein. Nuclei have low levels of Smad2-Venus fluorescence (A), but misexpression of the constitutively active TGF-β receptor Taram-D* results in strong nuclear Smad2-Venus fluorescence (B). (C and C′) Embryos at 6 hpf, previously injected with RNA encoding the VN and VC halves of Venus, pictured under transmitted (C) and UV (C′) light. Arrowheads highlight the nonfluorescent cells on top of the yolk (arrow). (D) Like Smad2-Venus transgenic embryos, the animal pole cells of embryos injected with RNA encoding VNSmad2 (VNS2) and VCSmad4 (VCS4) constructs are devoid of nuclear fluorescence. The arrow highlights intense nuclear fluorescence during cell division. (E) Misexpression of Taram-D* results in strong nuclear Smad2/4 BiFC. (F) Injection of RNA encoding a modified VNSmad2 construct that lacks the TGF-β SXS phosphorylation motif (VNS2ΔSXS) together with RNA encoding VCS4 and Taram-D* does not result in nuclear BiFC.

In a preliminary attempt to investigate nodal signalling levels during zebrafish development, we generated transgenic embryos that express a Smad2-Venus fusion protein under the control of a ubiquitous promoter. During zebrafish development, marginal cells are thought to receive the highest levels of endogenous nodal signalling while cells at the animal pole experience low levels, if any [14]. As predicted, nuclei of animal pole cells of transgenic embryos were only weakly fluorescent (Figure 1A), but expression of a constitutively active version of the TGF-β receptor Taram-A-D (Taram-D*) [15], caused strong nuclear fluorescence (Figure 1B). We note that in unstimulated cells, Smad2-Venus appeared to be concentrated at the centrosomes, and fluorescence accumulates in the nucleus shortly before nuclear envelope breakdown, only to disperse about a minute later, and then weakly associate with the mitotic apparatus (Video S1).

In an effort to improve the signal-to-noise ratio in such experiments, we turned to BiFC. When zebrafish embryos were injected with the N- and C- terminal halves of a modified form [12] of the fluorescent protein Venus [16], no fluorescence was observed, demonstrating that these fragments are suitable for BiFC experiments in this species (Figure 1C, C'). We therefore created fusions of the N- and C- terminal halves of Venus with the N termini of zebrafish Smad2 and Smad4, respectively, to create VNSmad2 and VCSmad4. When these constructs were expressed in the zebrafish embryo, *ntl* expression was unaffected in 90% of cases (*n* = 124), and in the remaining embryos, expression was normal in the marginal zone with weak ectopic expression in animal pole cells (unpublished data). These experiments demonstrate that our Smad BiFC constructs are suitable reagents for the analysis of endogenous nodal signalling.

Consistent with the experiments described above, we observed no nuclear BiFC fluorescence in animal pole cells of embryos injected with VNSmad2 and VCSmad4 (Figure 1D). As in the *Xenopus* embryo [12], however, and in contrast to the behaviour of Smad2-Venus, intense fluorescence appeared to be associated with chromosomes during cell division (Figure 1D, arrows, and Video S2). This is discussed below. When embryos received injections of both Smad BiFC constructs and the constitutively active version of Taram-A-D [15], strong nuclear fluorescence was observed in animal pole cells (Figure 1E). Similar results were observed when embryos were co-injected with RNA encoding our Smad2/4 BiFC constructs and the TGF-β ligand sqt (unpublished data). Activation of the TGF-β signalling pathway results in the phosphorylation of receptor-regulated Smads in their C-terminal SXS motifs [9]. Deletion of the SXS phosphorylation site in the VNSmad2 construct (VNS2ΔSXS) abolished TGF-β induced nuclear fluorescence (compare Figures 1E and 1F).

Together, these experiments demonstrate that our Smad2-Venus transgenic embryos and Smad BiFC constructs report the activation of the TGF-β signal transduction pathway in the zebrafish embryo.

### Visualisation of Endogenous Nodal Signalling

**Figure 2:**
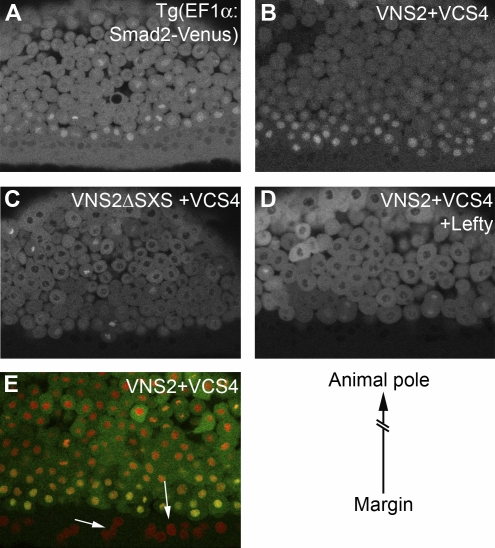
Visualisation of Endogenous Nodal Signalling Images of a Smad2-Venus transgenic embryo (A) and embryos injected with Smad2/4 BiFC constructs (B–E) are shown. Embryos are positioned such that marginal cells are at the bottom of each image, with animal pole cells towards the top (see diagram lower right). Smad2-Venus transgenic embryos (A) and embryos injected with the Smad2/4 BiFC constructs (B) display strong nuclear fluorescence in cells at the margin, while cells positioned nearer to the animal pole do not. (C) Nuclear fluorescence was not observed in the marginal cells of embryos injected with RNA encoding BiFC constructs that lack the TGF-β phosphorylation site (VNS2ΔSXS/VCS4). (D) Nuclear fluorescence in marginal cells was also abolished when embryos were co-injected with RNA encoding Smad2/4 BiFC constructs and the nodal antagonist Lefty (Antivin). (E) An embryo injected with RNA encoding Smad2/4 BiFC constructs (green) and a histone marker (red). Arrows show that nuclei in the YSL are devoid of Smad2/4 BiFC, while the most marginal nuclei show strong Smad2/4 BiFC and therefore appear yellow.

We first investigated endogenous nodal signalling in zebrafish embryos at 5–6 hours post fertilisation (hpf), when they express *ntl* and experience endogenous nodal signalling [17]. Observation of Smad2-Venus transgenic embryos at 6 hpf revealed a gradient of nuclear fluorescence that was high at the margin and decreased towards the animal pole (Figure 2A), indicating that there is a gradient of nodal signalling in the developing embryo. This impression was confirmed by use of Smad2/4 BiFC, where high levels of nuclear fluorescence were observed in marginal cells, with intensity gradually decreasing as distance from the margin increased (Figure 2B). This pattern of nuclear fluorescence was not observed in embryos injected with BiFC constructs lacking the TGF-β phosphorylation site (VNS2ΔSXS/VCS4) (Figure 2C), in embryos expressing the nodal antagonist lefty [4] (96%; *n* = 25) (Figure 2D), or in MZ*oep* embryos (100%; *n* = 15, unpublished data).

### Quantification of the Nodal Gradient

**Figure 3:**
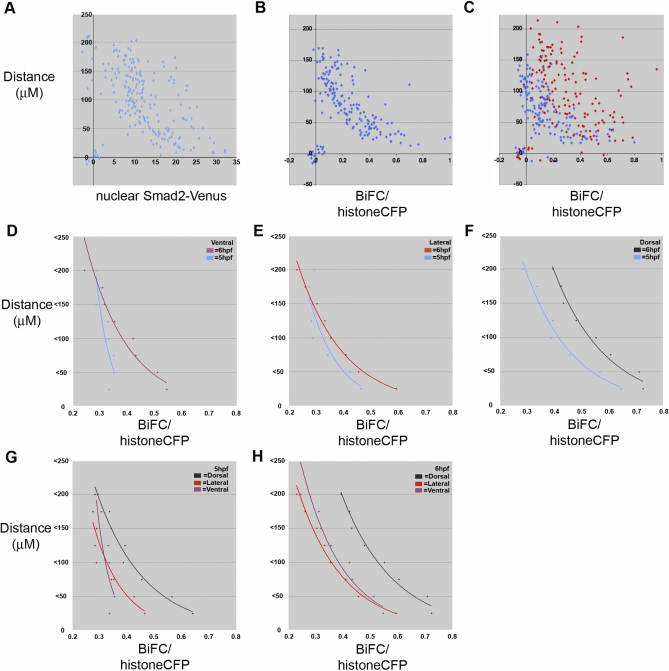
Quantification of Endogenous Nodal Signalling Plots of the nuclear Smad2-Venus fluorescence intensity in transgenic embryos (A, *x*-axis), or the ratio of nuclear Smad2/4 BiFC to histone CFP (B–H, *x*-axis), against distance from the margin (near zero) towards the animal pole (>200 μm) of the embryo (μm, *y*-axis). All graphs are plotted with the average distance and intensity of nuclei in the YSL as zero. (A) Nuclear Smad2-Venus fluorescence intensity in a 6 hpf transgenic embryo. (B) Nuclear Smad2/4 BiFC in a wild type 5 hpf embryo. (C) Comparison of Smad2/4 BiFC in lateral cells of the same embryo at 5 hpf (blue) and 6 hpf (red). (D–H) A comparison of the average BiFC intensity of all nuclei within 25-μm intervals of multiple embryos. (D–F) A temporal analysis of the average nuclear BiFC intensity in ventral cells (D), lateral cells (E), and dorsal cells (F) at 5 hpf (blue in all three) and 6 hpf. (G and H) A spatial analysis of nuclear BiFC at 5 hpf (G) and 6 hpf (H). As in (D–F), purple = ventral cells, red = lateral cells, and black = dorsal cells. The number of embryos used to calculate the average intensity for 5 hpf was: 7 ventral, 10 lateral, and 7 dorsal; numbers of embryos used for 6 hpf was: 5 ventral, 6 lateral, and 6 dorsal.

Smad2-Venus transgenic embryos do not exhibit detectable nuclear fluorescence in the yolk syncytial layer (YSL) of the embryo (Figure 2A), and nor do we observe Smad2/4 BiFC fluorescence in YSL nuclei of embryos co-labelled with a fluorescent histone marker (Figure 2E). These observations have allowed us to use Volocity software (Improvision) to quantify nuclear Smad2-Venus and Smad2/4 BiFC fluorescence intensity from the margin to the animal pole at different stages, defining the average intensity and average position of the YSL nuclei as zero (Figure 3A and 3B). We found that the most marginal nuclei, nearest the YSL, had the greatest Smad2-Venus fluorescence (Figure 3A) and the greatest Smad2/4 BiFC (Figure 3B). The nuclear fluorescence decreased in cells closer to the animal pole, some 200 μm away. Interestingly, nuclei positioned close to each other frequently had very different levels of nuclear Smad2-Venus and Smad2/4 BiFC fluorescence (Figure 3A and 3B; see also Figure 2A and 2B). One possibility is that these differences reflect local variation in effective nodal concentrations. Alternatively, there may be cell cycle–dependent variations in signal level associated with the intense fluorescence during cell division (Figure 1D, arrow, and Video S1).

We went on to investigate the spatial and temporal patterns of Nodal signalling by allowing embryos to continue development after imaging and then noting the positions of the imaged cells relative to the shield. This analysis exploited the superior signal-to-noise ratio of the Smad2/4 BiFC technique (see Figures 1 and 2). In preliminary experiments, analysis of lateral nuclei revealed that cells have higher levels of Smad2/4 signalling at 6 hpf compared to 5 hpf (Figure 3C; blue points are 5 hpf and red are 6 hpf). To improve our understanding of the spatio-temporal aspects of these signalling events, we calculated the average nuclear Smad2/4 BiFC intensity in 25-μm intervals from the margin towards the animal pole in several different embryos (Figure 3D–3H). We defined regions as dorsal, lateral, or ventral if the imaged cells were positioned within the dorsal quarter, lateral two quarters, or ventral quarter of the embryo, respectively. This analysis was performed for dorsal, lateral, and ventral cells at 5 and 6 hpf. As observed in individual embryos (Figure 3C), equivalently positioned cells have greater nuclear BiFC intensities at 6 hpf compared with 5 hpf, consistent with the idea that these cells experience increasing levels of nodal signalling during this period (Figure 3D–3F). When dorsal, lateral, and ventral cells were compared, we observed that lateral and ventral cells experience near identical levels of nodal signalling but dorsal cells experience higher levels (Figure 3G and 3H).

### Activation of Nodal Target Gene Expression

**Figure 4:**
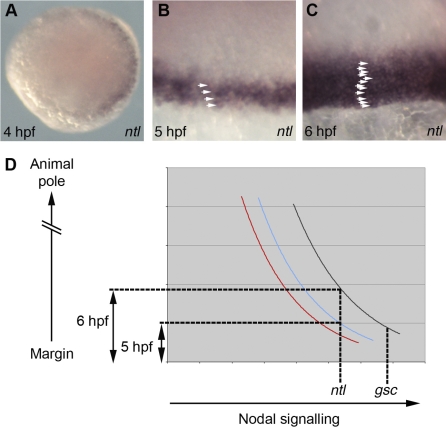
Spatial and Temporal Induction of Nodal Target Genes (A–C) Analysis of *ntl* expression. (A) Animal pole view of a 4 hpf embryo. (B and C) Views of the margin at 5 hpf (B) and 6 hpf (C) with arrowheads highlighting *ntl* expressing cells. (D) Graphical representation of Smad2/4 BiFC quantifications in Fig's 3E-F. Black: 6 hpf dorsal cells; blue: 5 hpf dorsal cells; red: 6 hpf lateral cells.

To place our observations in the context of normal development, we studied the expression profile of the nodal target gene *ntl* (Figure 4A–4C). *ntl* is first activated on the dorsal side of the embryo at 4 hpf (Figure 4A). Expression then spreads laterally, and by 5 hpf transcripts are detectable 3–5 cells deep throughout the margin (Figure 4B). By 6 hpf the *ntl* expression domain has doubled, and is now approximately 12–14 cells deep (Figure 4C). The expansion of the *ntl* domain observed over this period reflects the increasing levels of nuclear BiFC fluorescence and of Smad2/4 signalling (Figure 4D compare blue and black trend lines).

**Figure 5:**
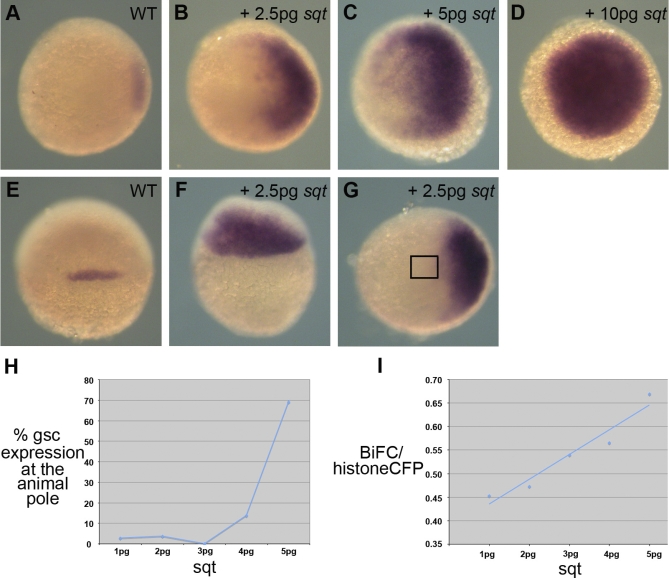
Levels of Nodal Signalling Specify Dorso-Ventral Pattern (A–F) In situ hybridisation showing expression of *gsc* in 6 hpf wild-type (WT) embryos (A and E), and embryos injected with increasing concentrations of *sqt* (B–D, F, and G). (A–D, and G) Animal pole views, with dorsal towards the right of the image, show that increasing concentrations of *sqt* lead to a ventral expansion of *gsc.* (E and F) Images of the dorsal side of (A) and (B) show that *gsc* expression expands towards the animal pole in embryos injected with *sqt*. (G–I) Correlation of the percentage of embryos that express ectopic *gsc* with levels of Smad2/4 nuclear BiFC. (G) No ectopic *gsc* expression is visible in the animal pole (boxed region) of an embryo injected with 2.5 pg of sqt mRNA. (H) Graph showing the percentage of embryos that express *gsc* in animal pole cells in response to increasing levels of *sqt* mRNA. Note that significant expression of *gsc* occurs between 4 pg and 5 pg of injected mRNA. (I) The average intensities of Smad2/4 nuclear BiFC in animal pole cells of embryos injected with the indicated amounts of *sqt* mRNA, using the same groups of embryos used to quantify *gsc* expression in (H). Smad2/4 BiFC intensities were normalised by subtracting from the data the average intensity of nuclei in the YSL, derived from Figure 3. Numbers of embryos used for the quantifications of *gsc* expression (H) were: 5 pg = 103; 4 pg = 58; 3 pg = 58; 2 pg = 112; 1 pg = 36. Numbers used for the quantification of Smad2/4 BiFC intensity (I) were: 5 pg = 20; 4 pg = 8; 3 pg = 14; 2 pg = 16; 1 pg = 6.

Our BiFC results show that the highest levels of Smad signalling occur at the dorsal side of the zebrafish embryo near the margin, where *gsc* is expressed (Figures 3G, 3H, and 4D, compare red and black trend lines). Consistent with this observation, work in zebrafish and *Xenopus* indicates that activation of *gsc* requires higher levels of nodal or activin-like signalling than are required to induce *Brachyury* [2,18]. These results suggest, in contrast to previous proposals [13], that a gradient of nodal signalling specifies the dorso-ventral axis of the zebrafish embryo. To explore this point in more detail, we expressed increasing amounts of sqt in the embryo. Our results showed that as levels of sqt increased, the domain of *gsc* expression expanded both animally and ventrally, as exogenously introduced sqt supplemented levels of the endogenous protein (Figure 5B-5D and 5F).

In an effort to correlate, in a quantitative manner, sqt signalling with Smad2/4 BiFC and *gsc* expression, we injected embryos at the one-cell stage with increasing amounts of *sqt* mRNA. At 6 hpf, we then measured Smad2/4 nuclear BiFC in the animal pole cells of some of the embryos and processed the remaining embryos for *gsc* expression. Injection of 1–4 pg of *sqt* mRNA resulted in an expansion of the *gsc* expression domain, but few embryos expressed *gsc* at the animal pole (Figure 5G, boxed area). Injection of 5 pg of *sqt* mRNA resulted in a significant increase in the percentage of embryos that expressed *gsc* in animal pole cells (Figure 5H), suggesting that the threshold for activation of *gsc* lies between 4 and 5 pg of *sqt* mRNA*.* Quantification of nuclear Smad2/4 BiFC fluorescence in the animal pole cells of injected embryos demonstrated that as the levels of *sqt* increased, so did fluorescence intensity (Figure 5I). Based on these data, our results indicate that the threshold for the activation of *gsc* expression is represented by a nuclear Smad2/4 BiFC intensity between 0.60 and 0.65 (Figure 5I). The only cells to experience endogenous levels of Smad2/4 BiFC that exceed this threshold are dorsal marginal cells (Figure 3H).

**Figure 6:**
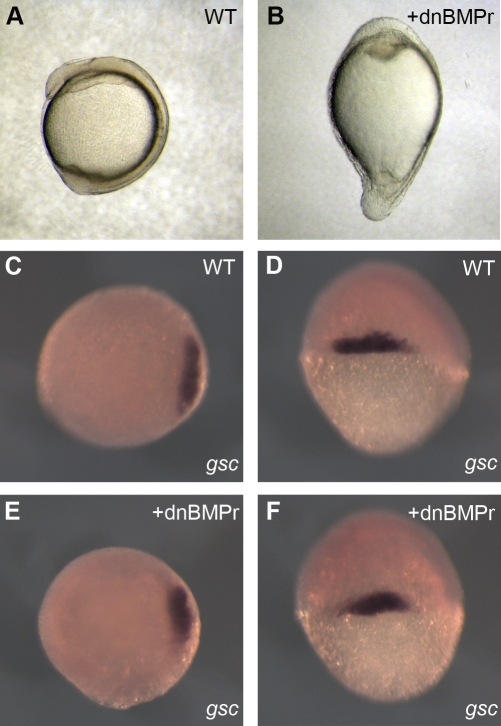
Dorso-Ventral Patterning Is Dependant on Nodal and BMP Signalling (A, C–D) Wild-type embryos. (B, E, and F) Embryos injected with 800 pg of RNA encoding a dominant negative BMP receptor. (A and B) Four-somite stage embryos. (C–F) *gsc* in situ hybridisation on 6-hpf embryos. (C and E) Animal pole views. (D and F) Dorsal views.

Our results are consistent with the idea that nodal signalling patterns the dorso-ventral axis of the zebrafish embryo as well as the animal-vegetal axis. But is nodal signalling the prime mover for dorso-ventral patterning in the zebrafish, or do sqt and cyc act downstream of BMP family members? Embryos lacking BMP signalling become dorsalised and fail to form ventral tissues [19], so it is possible that the dorso-ventral axis is first established by the ventral activation of the BMP signal transduction pathway, and it is this that directs the spatial distribution of nodal signalling and the dorsal activation of genes such as *gsc.* In this model, all dorso-ventral patterning would depend on BMP signalling, so to address the idea we injected embryos at the one-cell stage with a dominant negative BMP receptor (dnBMPr) and then studied the expression of *gsc.* Injected embryos became elongated (Figure 6A and 6B; 190/203 elongated at 4-somite stage) and appeared strongly dorsalised [20]; by 24 hpf almost all had died, with the survivors displaying slightly weaker dorsalised phenotypes (9 = c4, 9 = c3, and 7 = c2). *gsc* expression was unaffected in embryos injected with RNA encoding dnBMPr (Figure 6C–6F), indicating that the establishment of dorso-ventral patterning and the spatial distribution of nodal signalling is independent of BMP signalling.

## Discussion

Our understanding of the role of morphogen gradients during development is based largely on experiments that monitor gene expression after an increase or decrease in the concentration of a putative morphogen. This approach has the benefit of simplicity, but it is difficult to infer from the results obtained the shape of a morphogen gradient or the dynamics of its formation, because different genes respond differently to different morphogen concentrations, and because there may be interactions between gene products that refine their expression domains [21]. As an alternative, it is possible to observe the behaviour of tagged morphogens, including members of the TGF-β family [22–24], but these may not reflect accurately the behaviour of the endogenous inducers, and it is also possible that effective gradients of inducers are created by inverse gradients of an inhibitor [25,26]. We addressed these problems by using BiFC [11], a technique that permits quantitative assessment of levels of nodal signalling [12]. Our results demonstrate that the early zebrafish embryo experiences a gradient of nodal signalling levels, with cells at the margin experiencing the highest levels of nodal signalling and cells positioned away from the margin and towards the animal pole experiencing lower levels. Highest levels of signalling are experienced by cells at the dorsal margin of the embryo, where *gsc* is expressed.

### Nodal Signalling and Dorso-Ventral Patterning

Our results are consistent with the idea that nodal signalling patterns the animal-vegetal axis of the zebrafish embryo, with changes in the distribution and intensity of Smad signalling being reflected in changes in the spatial expression pattern of the nodal target gene *ntl* (Figure 4). In addition, we observe that over expression of *sqt* causes the expression domain of *gsc* to extend towards the animal pole (Figure 5E and 5F).

However, in contrast to previous conclusions based on cell lineage and gene expression experiments [13], our data also suggest that nodal signalling plays a role in patterning the dorso-ventral axis of the zebrafish. In particular, we note that there are higher levels of Smad2/4 BiFC fluorescence in dorsal regions than in lateral and ventral regions (Figures 3G, 3H, and 4D) and that *gsc*, whose expression requires higher levels of nodal signalling than does *ntl* [2], is expressed in these regions of elevated fluorescence. Consistent with this model, our correlation of Smad2/4 BiFC intensity with ectopic *gsc* expression (Figure 5H and 5I) demonstrates that the only cells to go above the *gsc* threshold are dorsal marginal cells. In addition, we note that over half of the cells of the prospective endoderm, a tissue whose formation also requires high levels of nodal signalling, are located dorsally [27].

If high levels of nodal signalling are indeed required for dorsal fates and lower levels for lateral and ventral tissues, then increased nodal signalling should produce a ventral shift in dorsal fates and loss or attenuation of nodal signalling should result in a dorsal shift of ventral fates. Consistent with this model, increased nodal signalling expands the expression domain of *gsc* in a ventral direction (Figure 5A–5D) and loss of nodal signalling results in a dorsal shift of the ventral marker *gata2* [5]. Similarly, fate mapping experiments demonstrated that cells fated to become pronephros and midbrain, which in wild-type embryos are located in ventral and lateral positions respectively, shift towards the dorsal side of *sqt-/-;cyc+/-* embryos [13]. However, some ventral markers, such as *spt* and *vox*, are not expanded dorsally in embryos with reduced nodal signalling [13]. It is likely that these genes are regulated by BMP family members [5]; if BMP signalling is attenuated, ventral tissues fail to form and embryos become dorsalised [19]. Significantly, we found that the expression of a dominant negative BMP receptor had no effect on the expression of *gsc* (Figure 6C–6F)*.* This suggests that the elevated levels of nodal signalling at the dorsal side of the embryo occur independently of BMP signalling. Previous work has demonstrated that Wnt/β-catenin signalling is also required for the specification of dorsal cell fates and that ectopic activation of β-catenin induces the expression of *gsc* [13,28]*.* However, *sqt* is not expressed in embryos with disrupted β-catenin signalling, and β-catenin cannot induce *gsc* expression in *sqt* mutant embryos [13,28]. Together with our correlation of nodal signalling and *gsc* expression, these results indicate that the effects of β-catenin are mediated by nodal signalling.

In combination with the results described above, our data therefore indicate that patterning of the zebrafish dorso-ventral axis involves high levels of BMP signalling in ventral tissues and high levels of nodal signalling in dorsal regions, effectively setting up a double gradient. It is also possible, as in the *Xenopus* embryo [29], that BMP ventralises the embryo only after the onset of gastrulation.

### Spatial and Temporal Induction of *ntl* Expression

As discussed above, the dynamic expression pattern of *ntl* (Figure 4B and 4C) reflects the spatial changes in nodal signalling that occur in the margin of the zebrafish embryo between 5 and 6 hpf (Figure 4D). Expression of both *cyc* and *sqt* declines between 5 and 6 hpf [13], so it is likely that the increased level of signalling experienced by cells positioned away from the margin at 6 hpf derives from nodal ligand that has traversed cell tiers 1–6 during this period.

At 6 hpf, Smad signalling extends farther towards the animal pole in dorsal regions of the embryo than in lateral and ventral regions (Figure 3H), yet *ntl* is expressed in approximately the same number of cell tiers throughout the margin, and does not spread into prospective neural tissue at the dorsal side [30]. This suggests that *ntl* expression is repressed in the prospective neural plate, perhaps, as in *Xenopus*, in a *Sip-1*–dependent manner [31,32]. It is possible that the Smad signalling that occurs in the neural plate provides positional information to this tissue; injection of increasing concentrations of lefty results in the gradual loss of hindbrain structures, whereas prospective forebrain tissues are converted into hindbrain structures following expression of *cyc* [4]*.*

### Why Is Nodal Signalling Higher in Dorsal Positions?

At 5 hpf, the expression of *sqt* and *cyc* is uniform throughout the margin of the zebrafish embryo [13], so what might cause the activation of Smad signalling to be higher in dorsal regions? Evidence suggests that the duration of signalling as well as the concentration of the morphogen may determine cell fate [17,33,34], and it may be significant that expression of sqt both commences on the dorsal side of the embryo and persists for longer in this region [13]. The elevated level of Smad2/4 BiFC in dorsal regions may therefore reflect both signal intensity and signal duration in the developing embryo.

### Subcellular Localisation of Smad2 and Complexes of Smad2 and Smad4

Our observations of transgenic embryos expressing Smad2-Venus indicate that Smad2 is associated with the centrosome, and comparison with results obtained with Smad1 [35] suggest that this might represent Smad2 that is destined for degradation. This is under investigation. We also noted that Smad2-Venus entered the nucleus shortly before nuclear envelope breakdown, and in this respect, its behaviour resembled that of cyclin B1, which translocates to the nucleus after phosphorylation by Polo-like kinase 1 [36–38]. We do not yet know if the translocation of Smad2-Venus is regulated by phosphorylation, but if it were, this newly phosphorylated Smad2 might then be able to associate with Smad4 and form a complex on the chromosomes. We do not understand the significance of such an association, although one possibility is that it ensures an equal distribution of Smads between daughter cells, as is thought to occur for Sara-containing endosomes in the developing fly wing [39].

## Materials and Methods

### Generation of transgenic embryos.

The Smad2-Venus fusion was generated by PCR amplification of Venus and cloning into a pCS2-Smad2 plasmid, thus generating a fusion of Venus to the N terminus of Smad2. The Smad2-Venus fusion was then subcloned into a miniTol vector containing the *Xenopus* EF1α, mcFos promoter. Transgenic embryos were generated by injecting embryos at the one-cell stage with 15 pg of Smad2-Venus miniTol plasmid and with 12.5 pg of transposase RNA. Injected embryos were raised to adulthood and then outcrossed to generate stable transgenic lines.

### Constructs and manipulation of embryos.

All constructs were injected in volumes of 2 nl into the yolk of zebrafish embryos at the one-cell stage, and embryos were then incubated at 28 °C. Where stated, embryos were injected with 100 pg of histone CFP [12], 50 pg of VNSmad2, 50 pg of VCSmad4, 300 pg of Lefty (Antivin) [4], 1 pg of Taram-A-D, 2.5–10 pg of *sqt* [2], or 800 pg of truncated dominant negative BMP receptor [40]. Zebrafish Smad2 and Smad4 open reading frames were amplified by PCR, cloned into the BiFC constructs [12], and sequenced. The VNS2ΔSXS construct was created by introducing a stop codon into the VNS2 plasmid using PCR based mutagenesis with the primers 5′-TTAGGACATACTTTAGCAGCGTACGGAGGGGGAGCCCATC- 3′ and 5′-GATGGGCTCCCCCTCCGTACGCTGCTAAAGTATGTCCTAA-3′. All RNA was synthesised using SP6 mMessage mMachine according to the manufacturer's instructions (Ambion). Whole mount in situ hybridisation was performed essentially as described [41], using probes specific for *ntl* [30] and *gsc* [42].

### Imaging and quantification.

For imaging, embryos were de-chorionated and embedded in 0.3% agarose. Images were obtained with Perkin Elmer spinning disc and Olympus FV1000 inverted confocal microscopes using 40× lenses. All quantifications were performed by sequential imaging of CFP and Venus fluorescence using the Olympus FV1000 microscope. Ten 1-μm Z sections of the cells nearest the lens (based on focal plane) were imaged. Following imaging embryos were incubated at 28 °C until 6–7 hpf. The agarose dish was then placed in hot water to melt the agarose, the embryos were removed from the agarose using forceps, and the positions of the imaged cells in relation to the shield was noted. Individual Z sections were used for the quantification of animal pole cells. Fluorescence intensity was quantified using Volocity software (Improvision). Individual nuclei were identified using a protocol to mark objects with intensities between 10 and 100% in the CFP (histone) channel. Quantifications were analysed using Microsoft Excel. For each image, the nuclei of the YSL were identified and the average distance and intensity of these nuclei was subtracted from all nuclei in that image. Video S1 was made using the Perkin Elmer spinning disc microscope.

## Supporting Information

Video S1 A Movie of a Smad2-Venus Transgenic Embryo Showing Fluorescence during Mitosis (23.19 MB AVI)

Video S2 A Movie of an Embryo Injected with the Smad2/4 Constructs Highlighting the Intense Fluorescence That Occurs during Mitosis (40.67 MB AVI)

## Abbreviations

BiFC: bimolecular fluorescence complementation
hpf: hours post fertilisation
YSL: yolk syncytial layer
